# Simultaneous noninvasive quantification of redox and downstream glycolytic fluxes reveals compartmentalized brain metabolism

**DOI:** 10.1126/sciadv.adr2058

**Published:** 2024-12-20

**Authors:** Saket Patel, Paola Porcari, Elizabeth Coffee, Nathaniel Kim, Marjan Berishaj, Thasin Peyear, Guannan Zhang, Kayvan R. Keshari

**Affiliations:** ^1^ Department of Radiology, Memorial Sloan Kettering Cancer Center, New York, NY, USA.; ^2^ Molecular Pharmacology Program, Memorial Sloan Kettering Cancer Center, New York, NY, USA.; ^3^ Department of Neurology, Memorial Sloan Kettering Cancer Center, New York, NY, USA.; ^4^ Weill Cornell Medical College, New York, NY, USA.

## Abstract

Brain metabolism across anatomic regions and cellular compartments plays an integral role in many aspects of neuronal function. Changes in key metabolic pathway fluxes, including oxidative and reductive energy metabolism, have been implicated in a wide range of brain diseases. Given the complex nature of the brain and the need for understanding compartmentalized metabolism noninvasively in vivo, new tools are required. Herein, using hyperpolarized (HP) magnetic resonance imaging coupled with in vivo isotope tracing, we develop a platform to simultaneously probe redox and energy metabolism in the murine brain. By combining HP dehydroascorbate and pyruvate, we are able to visualize increased lactate production in the white matter and increased redox capacity in the deep gray matter. Leveraging positional labeling, we show differences in compartmentalized tricarboxylic acid cycle entry versus downstream flux to glutamate. These findings lay the foundation for clinical translation of the proposed approach to probe brain metabolism.

## Teaser

Metabolic imaging visualizes different redox compartments across the brain.

## INTRODUCTION

One of the most profound developmental evolutionary changes that separate humans from lower species is the substantial increase in size and complexity of the human brain. Decades of research have illuminated the intricate connections of cells in the brain, much of which is driven by metabolic pathways that operate both for neurotransmission and at a fundamental level for energy production (*1*, *2*). Different regions of the brain are known to use differential metabolism to facilitate brain function, although changes in these metabolic processes are difficult to visualize (*3*). Moreover, concomitant metabolic processes including the generation of reactive oxygen species (ROS) and the change in brain redox biochemistry are exceedingly challenging to quantify in the brain. In disease, changes in redox have been implicated from brain tumors to neurodegeneration, including oncogenic transformation of low-grade gliomas with mutant isocitrate dehydrogenase, a redox pathway consuming reduced form of NADP^+^ (nicotinamide adenine dinucleotide phosphate) (NADPH), and the putative pathogenesis of Alzheimer’s disease (*4*–*8*).

Although many metabolic methods have been pioneered in the past two decades, it remains challenging to access the metabolism of the brain using these predominantly destructive approaches (*9*). Traditional imaging approaches have been explored including positron emission tomography and magnetic resonance imaging (MRI) (*10*, *11*), but they suffer from the inability to discern a metabolic product from a substrate and lack the sensitivity to measure a real-time metabolic flux. Moreover, although animal models can shed light on potential mechanisms in the brain, they do not reflect the complexity of the human brain; therefore, translational methods are needed to truly understand beyond metabolic pool sizes and provide insights into metabolic fluxes in human brain biology.

Isotope tracing methods with high sensitivity ex vivo analytical analysis have provided a means to estimate the contribution of substrates to their respective metabolite pools, revealing a wealth of information about the origins of a nutrient (*12*). These methods, although, fall short in the ability to measure a rate given the need to repeat the sample in the same region in time. The ability to investigate metabolism in real time and quantify dynamic changes soon after a rapid infusion of an isotopically enriched substrate has been enabled by boosting the intensity of the magnetic resonance signal, achieved via hyperpolarization (HP) (*13*, *14*). HP MRI has revolutionized the field of biomedical imaging by providing a noninvasive way to visualize in vivo metabolic processes, which allow the identification of metabolic aberrations in pathogenesis. HP MRI provides crucial and clinically relevant information for early-stage detection of disease and monitoring the therapy response. However, the potential of HP MRI has been limited due to the lack of efficient HP methods for key substrates of interest. After nearly two decades of scientific research in the field of HP ^13^C magnetic resonance, HP ^13^C-pyruvate remains the only metabolic probe translated to humans for investigating brain biochemistry (*15*–*19*). Although novel molecules have been developed for use in preclinical models (*20*), their use has been limited by the physical and chemical properties of the substrate as well as the feasibility of the clinical translation of HP methods (*14*). To exploit the vast potential of HP MRI in biomedical imaging, an HP approach characterized by a substrate with an appropriately long ^13^C spin-lattice relaxation time (*T*_1_) and no associated toxicity, high ^13^C polarization, and feasibility of clinical implementation is required.

Oxidative stress is typically the result of an imbalance between the cells generation of ROS and its ability to reduce them. Changes in oxidative stress have been associated with oncogenesis (*21*), but it also plays a crucial role in the pathogenesis of chronic diseases, such as cardiovascular diseases, diabetes, and neurodegenerative diseases (*8*, *22*). Therefore, an efficient HP probe that allows for the noninvasive in vivo investigation of oxidative stress could provide valuable insights into its role in disease development and progression. Such a probe could aid in early disease detection and improve current cancer therapies targeting oxidative stress [i.e., vitamin C (VitC) cancer therapy and radiation therapy] (*23*, *24*). Dehydroascorbic acid (DHA), which is the oxidized form of VitC, has been shown to rapidly transport into cells via the SLC2A family of solute carriers, predominantly SLC2A1 (GLUT1) (*25*). Hyperpolarized DHA can report on the redox state in vivo and act as a specific probe to assess oxidative stress in vivo (*26*). Conversion of DHA to VitC is a sensitive indicator of oxidative stress, making DHA an appropriate biomarker capable of differentiating between the oxidative stress in pathogenic and healthy tissues (*27*). However, ^13^C-DHA suffers from associated toxicity and a poor achievable ^13^C polarization due to its solubility in nonbiocompatible organic solvents (*28*). With several promising ongoing clinical trials targeting or leveraging oxidative stress for response, gaining key insights into DHA/VitC metabolism not only can improve the current understanding of the role of oxidative stress in pathogenesis but also has the potential to improve the development of therapies.

To address this, a previously unidentified hyperpolarized formulation of DHA leveraging its monomeric form was developed. The polarization of ^13^C-DHA was drastically improved by the newly created, clinically translatable formulation, which uses removable trityl radical, and has no associated toxicity. When using DHA as a solvent, the ability to codissolve it with pyruvic acid (PA) was demonstrated by establishing a simultaneous copolarization approach to monitor fluxes through both pathways simultaneously in the murine brain. This approach was extended to the copolarization of several endogenous substrates, making it a vehicle for the translation of other crucial HP substrates. To demonstrate the utility of such an approach, the metabolic flux of HP DHA and pyruvate was simultaneously probed, measuring metabolic flux rates in vivo. These fluxes were subsequently spatially resolved, and differential metabolic fluxes across regions of the murine brain were measured. To strengthen the value of the proposed HP approach, a xenograft mouse model of glioblastoma was investigated to evaluate the differential metabolism of the tumor lesion and the contralateral healthy tissue. Furthermore, the extension of this approach was demonstrated by spatially measuring multiple stages of pathway flux in the murine brain using multiple isotopic positions simultaneously, thus providing insights into the time-dependent compartmentalization of brain biochemistry.

## RESULTS

### Monomeric formulation of dehydroascorbate facilitates enhanced HP and copolarization with multiple substrates

**Fig. 1.**
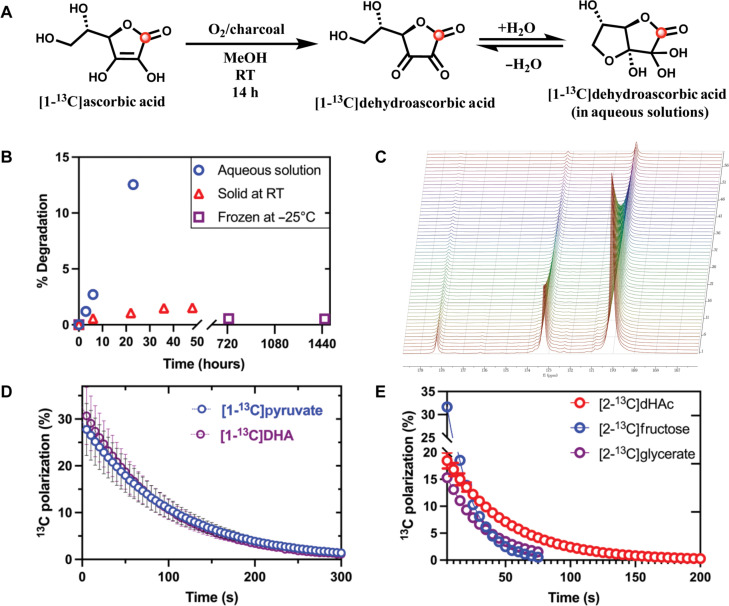
Chemistry of the [1-^13^C]dehydroascorbate. (**A**) Synthetic scheme of the [1-^13^C] DHA monomer and hydrated form of DHA in aqueous solution. h, hours. (**B**) Stability of the DHA monomer at room temperature (RT; red triangle), in aqueous solution (blue circle), and at −25°C (purple square). (**C**) ^13^C NMR dynamic spectra of hyperpolarized [1-^13^C]pyruvate (171.1 ppm) and [1-^13^C]DHA formulation doped with 15 mM AH111501 trityl radical. Samples polarized at 5 T and 0.8 K, dissolved in D_2_O, and neutralized by 4 M NaOAc in D_2_O. (**D**) Longitudinal ^13^C spin-lattice relaxation time, ^13^C *T*_1_, of HP [1-^13^C]pyruvate/[1-^13^C]DHA (6/4; v/v at 1 T) shown as a function of time and ^13^C polarization. (**E**) ^13^C polarization and ^13^C *T*_1_ of [2-^13^C]dihydroxyacetone (DHAc; red circle), [2-^13^C]glycerate (purple circle), and [2-^13^C]fructose (blue circle) in aqueous DHA at 1 T.

Conventionally, the bench-stable dimeric form of DHA has been used for studying the properties and activity of DHA using HP ^13^C magnetic resonance spectroscopy (MRS). However, the dimeric form of DHA is not readily soluble at high concentrations in physiological or aqueous solvents. The poor solubility of the DHA dimer in its standard formulation leads to a poor achievable ^13^C polarization (≤7%), making the assessment of the in vivo metabolic transformation challenging (*26*). The DHA dimer is chemically synthesized from the oxidation of ascorbic acid, and the synthesis of the DHA dimer goes through the DHA monomer as an intermediate compound, which is then converted to the bench-stable DHA dimer because the monomeric form of DHA has been perceived as being unstable. The limitations of the dimeric form were addressed by pursuing the possibility of generating a monomeric formulation. In our approach, the reaction intermediate, DHA monomer, was isolated as a colorless semisolid (Fig. 1A) and its stability investigated (Fig. 1B and fig. S3). At room temperature, degradation of the DHA monomer was observed after 3 hours when in aqueous solution, whereas it was less than 2% up to 4 hours for solid DHA (Fig. 1B). When DHA was kept stored at −25°C, no degradation was observed for up to 2 months (Fig. 1B). Although the DHA monomer is highly soluble in water (up to 8 M), it was dissolved with neat PA in 60/40 (PA/DHA; v/v), achieving a homogeneous glass-forming formulation, which resulted in high liquid-state polarization for both DHA and pyruvate (~30% for both molecules) with no change in ^13^C *T*_1_ (Fig. 1D). Typically, a neat PA sample polarizes to ~35% on our current system; the slight reduction in achieved polarization could be attributed to the dilution of PA from neat (14 M) to 8.8 M in the reported formulation. This approach was further extended to the combination of [1-^13^C]pyruvate and [2-^13^C]pyruvate with [1-^13^C]DHA, finding no loss in performance (fig. S4C). Given the planar geometry of DHA, we hypothesized that, in this neat formulation, it would be amenable to many chemical structures, providing a unique solvent to facilitate efficient polarization transfer. To this end, a range of chemical space was pursued to assess the ability of DHA to be used as a dynamic nuclear polarization (DNP) solvent (Fig. 1E and table S1). Monomeric DHA was found able to solubilize several molecules of biochemical importance to a higher concentration than previously described (table S1). Moreover, it facilitates significantly higher polarizations than those achieved previously under comparable polarization conditions, thus enabling the simultaneous metabolic investigation of the substrate along with DHA (*29*–*33*).

### Dynamic HP ^13^C MRS measures quantitative metabolic fluxes derived from HP DHA and HP pyruvate simultaneously in vivo

**Fig. 2.**
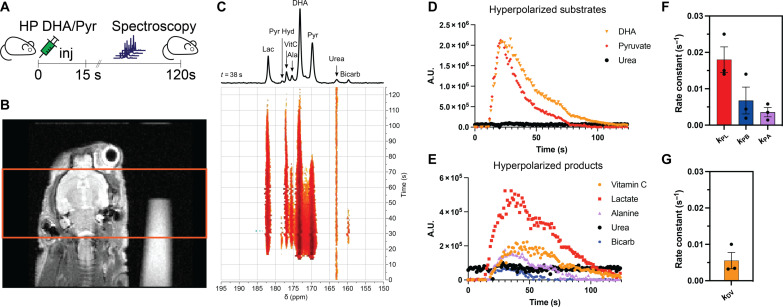
Dynamic HP MRS of HP [1-^13^C]pyruvate/[1-^13^C]DHA reveals rapid metabolic flux in the murine brain. (**A**) Schematic diagram of the experimental timeline and setup. (**B**) Axial view of a mouse brain with accompanying slice selective excitation (15 mm in thickness covering the murine brain and the urea phantom) used for ^13^C MRS in vivo. (**C**) Evolution of metabolic substrates and their metabolic products observed in a murine brain after injecting 300 μl of cohyperpolarized [1-^13^C]pyruvate and [1-^13^C]DHA in D_2_O in a nude mouse. ^13^C NMR spectra, acquired on a murine brain with a temporal resolution of 1 s, show the dynamics of both [1-^13^C]DHA and [1-^13^C]pyruvate along with their metabolic products. Downstream metabolites derived from pyruvate (lactate, pyruvate hydrate, alanine, and bicarbonate) and DHA (VitC) are annotated on a representative ^13^C NMR spectrum acquired 38 s after injection. (**D**) Time course graphs of HP [1-^13^C]pyruvate (red, rhombus) and [1-^13^C]DHA (orange triangle), along with their metabolic products (**E**). A.U., arbitrary units. The [1-^13^C]urea signal (black circle) of the phantom placed adjacent to the mouse, and used as a reference, is shown in (D) and (E). (**F**) Comparison between the calculated kinetic rate constants derived from the metabolic conversion of [1-^13^C]pyruvate to its products: [1-^13^C]lactate (*k*_PL_, red), [1-^13^C]bicarbonate (*k*_PB_, blue), and [1-^13^C]alanine (*k*_PA_, lavender). (**G**) Calculated kinetic rate constant deriving from the metabolic conversion of [1-^13^C]DHA to [1-^13^C]VitC (*k*_DV_, orange). Values are reported as means ± SE (*n* = 3 biological replicates per group).

Overcoming the limitations of previous attempts to trace the metabolism of HP DHA provides the means of studying the redox-mediated process in the murine brain. Using the proposed approach, wild-type mice were injected with an HP solution containing [1-^13^C]DHA and [1-^13^C]pyruvate, and dynamic ^13^C MRS was performed on the mouse brain (Fig. 2, A and B). Representative in vivo data demonstrate the delivery and conversion of both substrates to their known products (Fig. 2C). Kinetics of delivery demonstrates comparable time of arrival and sustained metabolic conversion (Fig. 2, D and E). With this enhanced sensitivity afforded by the increased polarization, the kinetic rates were fit for each of the metabolic fluxes allowing for the ability to compare these pathways in vivo. Given that they are in the same bolus, their direct comparison can be considered. Both *k*_PL_ and *k*_PB_ are defined as standard kinetic rates measured in the human brain. The measured rates in the combined formulation are comparable to previously published measurements of HP pyruvate (*15*, *18*, *19*) (Fig. 2F), whereas *k*_DV_ is in line with previous measurements in rat models of HP DHA (Fig. 2G) (*34*). These findings suggest that the conversion rate is adequate for in vivo detection in humans at the considered concentration and provide support for potential clinical translation of DHA as an HP imaging agent.

To further demonstrate the applicability of this approach, simultaneous injection of HP copolarized [1-^13^C]pyruvate and [1-^13^C]DHA was applied to a murine U87 glioblastoma model (fig. S6). Both substrates, [1-^13^C]pyruvate and [1-^13^C]DHA show slightly increased delivery to the tumor as expected from a previous work in rat models (fig. S6, C and D) (*35*). Although VitC levels were modestly increased in the tumor, a significant elevation in [1-^13^C]lactate was observed (fig. S6, E and F) analogous to previous reports. This further supports the ability to simultaneously measure these metabolic conversions in both the normal brain and tumor models, here in the mouse brain.

### Spatially resolved HP MRI reveals differential redox compartmentalization

**Fig. 3.**
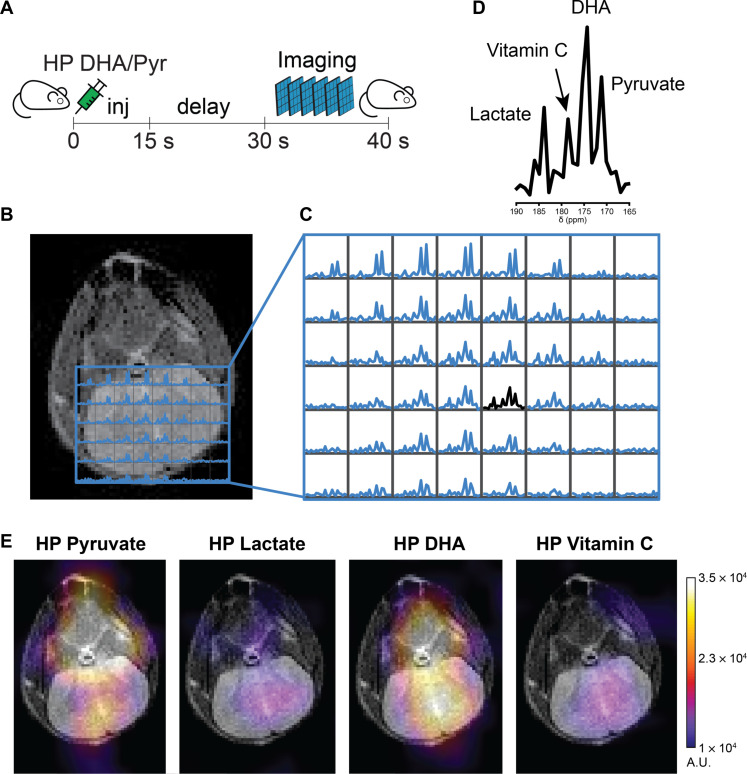
Simultaneous spatially resolved metabolic conversion of both HP DHA and HP pyruvate in the murine brain. (**A**) Scheme of the acquisition strategy, HP DHA/pyruvate injected over 10 s. After a delay of 15 s, a rapid multislice 2D EPSI acquisition was carried out, which covers the entire mouse brain. (**B**) Representative coronal slice of the murine brain with the annotated corresponding ^13^C MRSI grid from the stack of images acquired after infusion of HP DHA/pyruvate. (**C**) Expanded grid of ^13^C spectra demonstrating differential conversion of DHA and pyruvate in the slice. (**D**) Representative ^13^C spectrum annotating the peaks of interest, which is the same spectrum drawn in black from (C). (**E**) Representative color overlay maps of the area under the curve of each metabolite, demonstrating the distribution of HP substrates and products in the representative slice.

Although exciting as a redox probe, it has been challenging to assess the compartmentalization of these pairs in the murine brain in vivo. Leveraging this previously unknown approach, measurable differences in the gray matter (GM) and white matter (WM) for these fluxes were investigated. To determine these changes, a second cohort of mice was imaged using a two-dimensional (2D) multislice echo-planar spectroscopic imaging (EPSI) approach, allowing for the assessment of all metabolites throughout the brain (Fig. 3A). Representative ^13^C spectra demonstrate the ability to spectrally resolve the metabolites and visualize conversions throughout the brain (Fig. 3, B to D). Integrating these peaks allows for the generation of spatial maps for each metabolite in the simultaneous experiment (Fig. 3E).

**Fig. 4.**
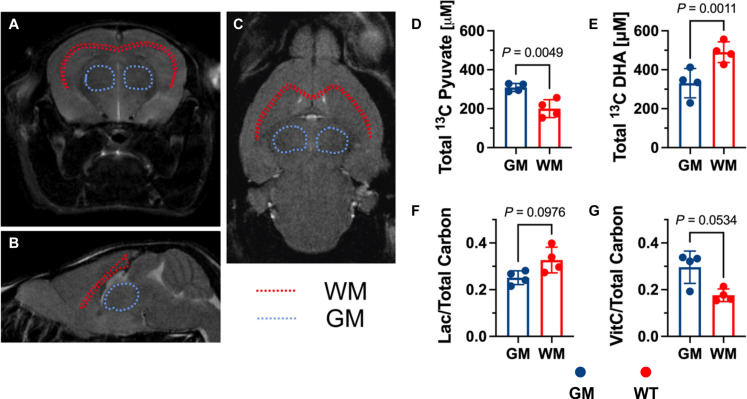
2D multislice EPSI for the quantification of metabolism in different brain compartments. (**A** to **C**) Representative T_2_-weighted ^1^H MRI of the murine brain in (A) coronal, (B) sagittal, and (C) axial planes annotating the segmentation of the WM (red dotted lines) and GM (ocean-blue dotted lines) for subsequent analysis. (**D** to **G**) Quantification of (D) total carbon derived from HP pyruvate (Total ^13^C Pyruvate), (E) total carbon derived from HP DHA (Total ^13^C DHA), (F) ratio of lactate/total carbon derived from pyruvate (Lac/Total Carbon), and (G) ratio of VitC/total carbon derived from DHA (VitC/Total Carbon) (*n* = 4 biological replicates per group).

Because data are multislice, they can be reconstructed into a 3D dataset and used to segment regions of GM and WM, providing a direct comparison of relative delivery and fluxes in both compartments (Fig. 4, A to C). By normalizing to the urea phantom (fig. S7), the concentrations of HP substrates and products were estimated in vivo (Fig. 4, D to G, and fig. S7, A to C). The calculated absolute concentrations shed light on the achieved substrate concentration in the brain for each molecule, given a bolus injection of them combined. These data would suggest that, although ~300 μM pyruvate and DHA are achieved in the GM, a much higher concentration of DHA is achieved in the WM (~500 μM). Given transport dynamics across the blood-brain barrier (BBB) and ultimate uptake in the cell, availability of the substrate locally in time will play an important role in understanding future metabolic fluxes. A relatively increased conversion of pyruvate to lactate was found in the WM, whereas the reverse was seen for DHA with increased VitC localized in the GM (Fig. 4, F and G).

### Copolarization of multiple isotope positions annotates spatially defined metabolic fluxes in the murine brain

**Fig. 5.**
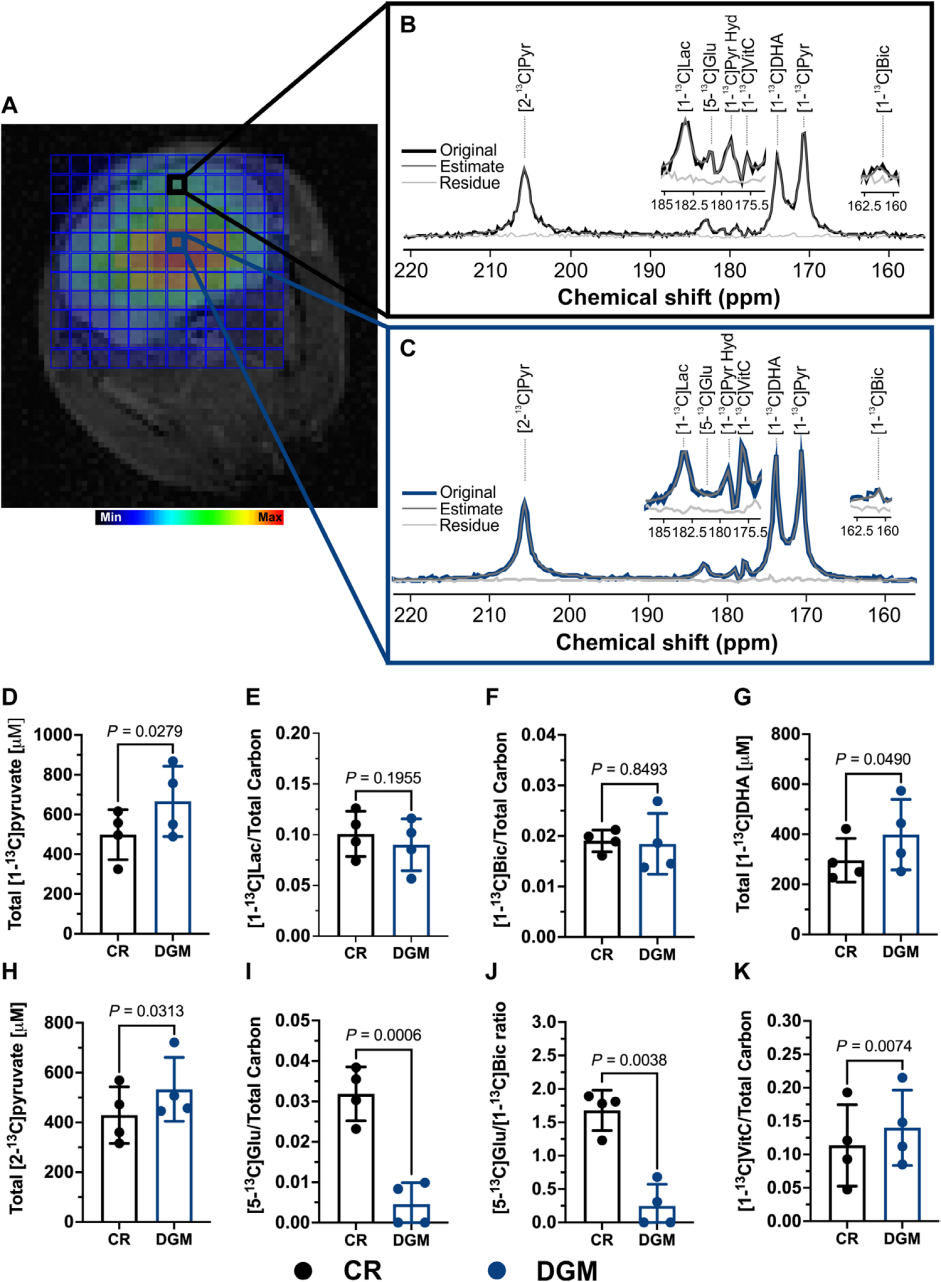
2D multivoxel CSI with HP [1-^13^C]pyruvate, [2-^13^C]pyruvate, and [1-^13^C]dehydroascorbate (DHA) to infer regional metabolism in a mouse brain. (**A**) Overlay of a coronal multivoxel ^13^C HP MRSI acquisition of a mouse brain in color levels (blue, min; red, max) on the corresponding morphological ^1^H T_2_-weighted image (in gray levels). The color of each voxel of the MRSI grid indicates the total ^13^C signal in that voxel. (**B**) Representative ^13^C HP spectrum (black line) of a voxel in the CR of a mouse brain showing the main metabolite peaks ([1-^13^C]pyruvate at 171.1 ppm, [1-^13^C]bicarbonate at 161.1 ppm, [1-^13^C]pyruvate-hydrate at 179 ppm, [1-^13^C]lactate at 183 ppm, [2-^13^C]pyruvate at 206 ppm, [5-^13^C]glutamate at 181.4 ppm, [1-^13^C]DHA at 174 ppm, and [1-^13^C]VitC at 178 ppm). (**C**) Representative ^13^C HP spectrum (blue line) of the DGM region of a mouse brain. For both spectra, a quantitative estimate (dark gray line) is shown along with the residual line (light gray line). A magnification of the spectral regions where the metabolites of interest are observed (175.5 to 185 and 160 to 162.5 ppm) is shown for each spectrum. (**D** to **F**) Quantification of total [1-^13^C]pyruvate delivered and ratios of its products lactate and bicarbonate to total carbon. (**G**) Quantification of total [1-^13^C]DHA. (**H** and **I**) Quantification of total [2-^13^C]pyruvate delivered and ratios of its products glutamate to total carbon. (**J**) Ratio of C5 glutamate to bicarbonate. (**K**) Ratio of [1-^13^C]VitC to total carbon derived from [1-^13^C]DHA (*n* = 4 biological replicates per group).

Regional differences in metabolic pools reflect the generation and accumulation of key metabolites in the brain. One such metabolic flux is the generation of glutamate in the brain from glycolytic flux to facilitate neurotransmission. To determine whether pyruvate utilization through multiple pathways along with DHA could provide insights into this compartmentalization, the copolarization of [1-^13^C]pyruvate, [2-^13^C]pyruvate, and [1-^13^C]DHA was used. To capture the formation of both ^13^C-bicarbonate from [1-^13^C]pyruvate and [5-^13^C]glutamate from [2-^13^C]pyruvate, imaging was performed within 15 s after injection (Fig. 5A). Moreover, to provide the necessary spectral resolution, a 2D chemical shift imaging (CSI) approach was used. As anticipated, all three products in the murine brain (bicarbonate, glutamate, and VitC) were visualized (Fig. 5, B and C). To further confirm that the resonance annotated and adjacent to lactate was C5 glutamate, complimentary in vivo experiments were conducted with only hyperpolarized [1-^13^C]pyruvate and [1-^13^C]DHA to demonstrate that the peak was undetected (fig. S8A).

Similar to the previous multislice experiment, both C1 and C2 pyruvate were similarly distributed throughout the brain. In contrast, the products C1 lactate and C1 VitC were respectively higher in the cortex and deep GM (DGM) brain regions. Using those metabolites as spatial landmarks, the distribution of other quantified metabolites could be compared. Unexpectedly, although bicarbonate was observed in both the cortex and DGM to a similar degree, C5 glutamate appears to only be significantly produced in the cortex region (Fig. 5, D to K). To determine what mechanistically drives these metabolic fluxes, matrix-assisted laser desorption ionization (MALDI) mass spectrometry imaging (MSI) (*28*, *29*) was conducted on murine brains (fig. S8, E to G) (*36*, *37*). By measuring glutamate and lactate pool sizes, a colocalization of these pools with the HP pyruvate–derived conversions was observed, suggesting these pool sizes as the result of metabolic flux in those areas. In contrast, VitC was accumulated to a much higher degree in the cortex, whereas the conversion of HP DHA to VitC occurred more markedly in the DGM (fig. S8B). Given the mechanism by which DHA is converted to VitC (*26*), cofactor availability that actually drives its conversion was postulated. The predominant cofactor for DHA reduction in the brain is glutathione (GSH). Moreover, MALDI MSI data revealed high GSH concentrations in the DGM (fig. S8C), suggesting that DHA was first converted to VitC in the GM and then potentially shuttled to other brain regions outside of the HP timescale. Together, this approach provides a means of annotating a completely different metabolic parameter, spatially defined metabolic flux, as compared to pool sizes measured by traditional methods.

## DISCUSSION

Given the putative direct link between bioenergetics and brain function as well as the dysregulation of these processes in disease, our understanding of this link in the brain is still poorly understood in humans (*1*, *2*). The regulation of brain redox along with the response to oxidative stress in different brain regions both implicated in several brain diseases (e.g., cancer and neurodegenerative diseases) requires new clinical translational methods to measure metabolic fluxes in vivo. Moreover, the degree of compartmentalized metabolism conducted by brain regions is of great interest. To this end, a previously unidentified approach capable of studying brain redox and glycolytic metabolism noninvasively was developed and used to characterize the spatial distribution of metabolic flux.

The rationale behind this study exploits the possibility of investigating different aspects of brain redox homeostasis through two reactions: the conversion of pyruvate to lactate and DHA to VitC. Both can be traced simultaneously in the brain when investigated with the two HP copolarized probes. Pyruvate and DHA cross the BBB via monocarboxylate transporters and GLUT1 transporters, respectively. Pyruvate is converted to lactate by LDH (lactate dehydrogenase) enzyme, which requires reduced form of NAD^+^ [nicotinamide adenine dinucleotide] (NADH) oxidation, whereas DHA is reduced to VitC through a reaction dependent on both GSH and NADPH. Therefore, quantification of the metabolites involved to both reactions provide insights into the redox status in the brain by simultaneously sampling both the NAD^+^/NADH and NADPH/NADP^+^/GSH pools. In the intact brain, comparable transport dynamics are seen for pyruvate and DHA, whereas in comparison to tumor-bearing mice with orthotopic xenografts, increased pyruvate delivery to the brain is observed with minimal change in DHA. This suggests that pyruvate transport is rate limited by the BBB and DHA transport is not. Differential transporter expression has been demonstrated in models of disease beyond cancer, for example, in loss of neuronal pyruvate dehydrogenase (*38*), making it extremely important for understanding brain biology. This differential transport dynamics is easily observed when the molecules are infused simultaneously, and future studies will be needed to tease out the instances where transport and metabolism are modulated in vivo.

Using this approach, it was feasible to spatially resolve brain compartments capable of driving more pyruvate reduction to lactate in contrast to regions where more DHA was reduced to ascorbate. Given the redox cofactors and mechanisms that drive each metabolic flux, this approach suggests that the DGM of the murine brain more significantly accumulates ascorbate on this timescale, likely as the site of increased reducing potential. In these brain regions, correlative MALDI MSI also demonstrated larger pool sizes of GSH, the cofactor predominantly responsible for DHA reduction. In contrast, a slightly higher lactate production was found in the murine cortex and WM. These findings are supported by recent translational human studies performed with hyperpolarized C1 pyruvate, where high levels of HP lactate were found in the normal appearing WM (*17*, *39*, *40*). However, these differences appeared to be minor (*19*) when data were normalized to pyruvate delivery (*19*).

Using three HP probes simultaneously, the differential pyruvate metabolism along with the DHA compartmentalization was quantified. It was observed that, whereas bicarbonate production resulting from the oxidation of HP C1 pyruvate, such as lactate, appears throughout the murine brain, C5 glutamate production resulting from either the oxidation or multiple tricarboxylic acid (TCA) cycle steps of HP C2 pyruvate predominantly occurs in the cortex although virtually absent in the DGM. The latter corresponds to those brain regions with high glutamate pool size, as shown in MALDI MSI data, suggesting that the metabolic flux through the TCA cycle to this pool is reflective of the brain regions that are responsible for high net flux to glutamate. This is well aligned with both recent studies using ^13^C isotope tracing in the murine brain as well as a recent HP C2 pyruvate study in humans that suggests localization to cortex (*41*). This highlights the nature of the proposed approach and the uniqueness of the data generated from real-time metabolic flux imaging. Like isotope tracing, this rapid HP MRI approach reveals where first-pass metabolic conversion happens in a spatial region, which may not be correlated to where a metabolite pool size is large. Moreover, given the rapid development of methods to spectroscopically image and encode HP metabolites throughout the brain, application of such approaches to humans could yield an unparalleled amount of human brain metabolic flux data in a single imaging session.

A limitation of the current study is linked to the acquisition strategy used and its parameters (e.g., in-plane nominal resolution and slice thickness) for both multislice EPSI and multivoxel CSI data acquisition. With standard preprocessing, including zero filling, the nominal reconstructed resolution was 1.3 x 1.3 mm^2^ (EPSI) and 0.9 x 0.9 mm^2^ (CSI). To best estimate the metabolite contribution to specific brain regions, high-resolution T_2_-weighted images were used for segmentation and pixels from the ^13^C data that were predominantly in the region of interest (ROI) were included in analysis to minimize partial volume effects. This, however, does not completely resolve the GM and WM of the mouse brain, and future studies using spectroscopic imaging approaches such as frequency specific excitation and EPI readouts will be needed to push the spatial resolution boundary to further investigate these regions. Considering these limitations, the findings of this study still provide trends that we believe will aid in the better understanding of the mouse brain and lay the foundation for other simultaneous approaches.

Further studies are necessary in humans to truly determine the regional distribution of these metabolic fluxes, but this work suggests the feasibility of such an approach and provides a formulation and methodology to conduct such clinical trials. Previous formulations of DHA were incompatible with clinical translation due to the solvent system used and the inability to generate a polarization state and dose necessary for human studies. The formulation achieved here overcomes all of these limitations, creating a neat DHA formulation. Future studies are aimed at translating this formulation to humans and the first measurements of redox in the human brain.

## MATERIALS AND METHODS

### Reagents

Isotopically enriched [1-^13^C]ascorbic acid, [1-^13^C]PA and [2-^13^C]PA along with deuterium oxide (D_2_O), sodium acetate, activated charcoal, and methanol (ACS reagent, >99.8%) were purchased from Sigma-Aldrich (St. Louis, MO, United States). Radical AH111501 was purchased from GE HealthCare (Chicago, IL, United States). All other reagents and solvents were used directly as supplied by the chemical manufacturers.

### Synthesis characterization

The DHA synthesis was carried out in oven-dried glassware. ^1^H and ^13^C nuclear magnetic resonance (NMR) spectroscopy was performed on DHA solutions in D_2_O using a Bruker AVANCE III 600 MHz (14.1 T) vertical bore NMR spectrometer (Bruker BioSpin, Billerica, MA, United States) at 25°C. NMR spectra of DHA solutions are described by the following nomenclature: δ = shift and multiplicity (s = singlet, m = multiplet, t = triplet, d = doublet, q = quartet, and dd = doublet of doublets) with coupling constants reported in hertz. The melting point was measured using the OptiMelt automated melting point system (Stanford Research System, Sunnyvale, CA, United States). The pH of aqueous solutions was measured using a pH meter (Thermo Fisher Scientific, Waltham, MA). NMR data were processed using the Mnova Software Suite (version 14.3.2, Santiago de Compostela, Spain).

### HP methods

One hundred microliters of [1-^13^C]DHA/[1-^13^C]PA (40/60; v/v) doped with 15 mM AH111501 trityl radical was prepared. The sample was polarized for 1.5 to 2 hours on a SpinLab hyperpolarizer (5 T, 0.8 K, GE HealthCare) by irradiating it at a 139.88-GHz microwave frequency. After polarization, the frozen sample was dissolved in 9.0 ml of D_2_O and pyruvate was neutralized by adding 216 μl of a 4 M sodium acetate solution (in D_2_O) to the dissolute to afford the HP solution of pH ~ 5.5. The HP solution contained 40 mM [1-^13^C]DHA and 90 mM [1-^13^C]PA. Approximately 700 μl of the HP solution was transferred to a 5-mm NMR tube and loaded to a 1-T Spinsolve ^13^C NMR spectrometer (Magritek, Wellington, NZ) for ^13^C NMR acquisition. NMR spectra were acquired with a flip angle excitation of 5° every 5 s over 4 min. The spin-lattice relaxation time (*T*_1_) was estimated by a monoexponential fit and corrected for the flip angle. Thermal polarization was determined from the average spectrum of 256 scans acquired with a flip angle of 90° every 10 s. Final polarization values, corrected for the flip angles and the apparent *T*_1,_ were 5 to 10% for all dissolutions. The concentration of ^13^C-DHA and ^13^C-PA in the HP solution was measured by ^13^C NMR at 600 MHz in the presence of 6 μl of 1 mM Gd-DOTA and 100 μl of 100 mM [1-^13^C]lactate standard added to 500 μl of the HP solution.

### Animals

All animal experiments were conducted under the approval of the Institutional Animal Care and Use Committee of Memorial Sloan Kettering Cancer Center in accordance with the standards for animal care and use, as set by the federal law under the Animal Welfare Act (13-12-019). Eight female athymic nude mice (the Jackson Laboratory, Bar Harbor, ME, United States) aged 16 to 20 weeks and weighting 28 to 36 g and four female C57BL/6J mice (the Jackson Laboratory) aged 22 weeks and weighting 30 to 37 g were used in this study. For tumor imaging, three female athymic Foxn1nu mice (Charles River, Wilmington, MA, United States) aged 8 to 10 weeks and weighting 22 to 25 were investigated. Mice were housed in groups of up to five mice per cage with free access to food and water, a 12-hour:12-hour light:dark cycle, temperature of 18° to 23°C, and humidity of 40 to 60%. Before imaging, each mouse underwent tail vein cannulation using a 23-gauge rodent tail-vein catheter (Braintree Scientific, Braintree, MA, United States) filled with heparin (1 U/ml; Fresenius Kabi, Lake Zurich, IL, United States) in 0.9% of sodium chloride. Anesthesia was performed with inhaled isoflurane mixed with oxygen delivered at a rate of 0.5 to 1 liter/min. Isoflurane concentration was 3% during induction (2 min) and 1.0 to 2% through acquisition. Anesthetized mice were laid prone on a dedicated holder that ensured minimal motion during scans. Respiration rate and body temperature were monitored through an MR-compatible system (Model 1025, SA Instruments Inc., Stony Brook, NY, United States) and maintained at 85 ± 20 breaths/min and 37° ± 0.5°C by regulating the isoflurane concentration and circulating warm water system, respectively.

Five minutes before the injection of the cohyperpolarized ^13^C probes, the isoflurane flow was turned off to perform the hyperpolarized scan on the awake mouse. Immediately after the hyperpolarized scan, the anesthetic regime was restored until the end of the MRI session.

### Glioma model

#### Cell line

Human U87 glioblastoma cell line (American Type Culture Collection, Manassas, VA, United States) was used for orthotopic xenografts in nude mice. Cells were cultured in Dulbecco’s modified Eagle’s medium supplemented with 10% fetal bovine serum (Gibco BRL, Thermo Fisher Scientific), penicillin (50 U/ml) and streptomycin (250 μg/ml; Gibco) in a humidified 5% CO_2_/95% air at 37°C. Cells were split when reaching ~70% confluence and used until passage 10.

#### Tumor implantation

Orthotopic human gliomas were implanted in three athymic Foxn1nu mice. Anesthetized mice were placed into a stereotaxic frame (Stoelting Co., Wood Dale, IL, United States), and 1 × 10^5^ cells dissociated into a single-cell suspension were injected in a volume of 2 μl of phosphate-buffered saline via a stereotaxic injector at a flow rate of 2 μl/min (Quintessential Stereotaxic Injector) into the right striatum of the mice (coordinates from bregma: 0.0-mm posterior, 2.0-mm lateral, and 3.0-mm ventral).

### In vivo MRI and spectroscopy

Experiments were performed on a Bruker BioSpec 3.0-T/18-cm horizontal bore system (Bruker BioSpin, Billerica, MA, United States) equipped with a high-performance gradient set (max gradient strength: 900 mT/m; slew rate: 4200 mT/m per s). A dual-tuned ^1^H/^13^C transmit/receive radio frequency volume coil (30 mm in inner diameter; Bruker BioSpin MRI GmbH, Ettlingen, Germany) was used. After collecting scout images to assess the position of the mouse brain at the magnet isocenter, multislice T_2_-weighted fast spin echo (TSE) images of mouse brain were acquired in the coronal orientation as an anatomical reference [repetition time (TR)/effective echo time (TE_eff_) = 2500/33 ms, echo train length = 8, field of view = 32 × 32 mm^2^, matrix size = 128 × 128, in-plane resolution of 250 × 250 μm^2^, 21 slices of 1 mm in thickness covering the whole mouse brain, two averages, and scan time of 100 s]. Static magnetic field homogeneity was optimized using a localized field-map shimming routine (ParaVision 360, v2.0 pl 1, Bruker BioSpin), resulting in water linewidths of 15 to 23 Hz over the selected volume of the mouse brain. The hyperpolarized ^13^C MRSI scan was acquired on awake mice 25 s after starting the intravenous injection (10-s duration) of the cohyperpolarized ^13^C probes (details on the MR sequence used in each specific experiment are reported below). For quantification purposes, a cylindrical phantom (radius = 0.5 cm; height = 3 cm) containing a 6 M (unless specified) ^13^C-labeled urea solution was placed adjacent to the mouse brain, included in the field of view of T_2_-weighted and ^13^C MRSI scan.

#### In vivo ^13^C NMR spectroscopy

To trace, in real time, the metabolic flux in a mouse brain through hyperpolarized ^13^C MRS, three independent experiments were performed on athymic nude mice (*n* = 3). Mice were intravenously injected over 10 s with 300 μl of cohyperpolarized 40 mM [1-^13^C]DHA and 100 mM[1-^13^C]pyruvate. Following the mouse setup as described above, a ^13^C slice-localized single-pulse-and-acquire sequence (128-μs block pulse at a 15° flip angle, 10-kHz bandwidth, 5-kHz spectral width, 2048 points, one average, 240 repetitions, TR = 1000 ms, 15-mm slice thickness to cover the whole brain, and scan time of 4 min) was used. To monitor the evolution of the metabolic products and substrates in the mouse brain ([1-^13^C]pyruvate at 171.1 parts per million (ppm), [1-^13^C]bicarbonate at 161 ppm, [1-^13^C]alanine at 176 ppm, [1-^13^C]DHA at 174 ppm, and [1-^13^C]VitC at 178 ppm), the start of the dynamic ^13^C MRS scan was set immediately after the dissolution DNP of the hyperpolarized substrates and 10 to 15 s before injection of the hyperpolarized ^13^C probes. For each experiment, a cylindrical 4 M urea phantom (163 ppm) was placed adjacent to the mouse brain as a reference for the ^13^C signal normalization.

#### In vivo ^13^C EPSI

When studying the regional differences of metabolite conversion in a mouse brain, four independent experiments were performed on C57BL/6J (*n* = 4) mice. Following the mouse positioning and shimming optimization, mice were intravenously injected with 250 μl of cohyperpolarized 40 mM [1-^13^C]DHA and 100 mM [1-^13^C]pyruvate for 10 s. The ^13^C hyperpolarized scan was acquired in the coronal orientation of the mouse brain using a multislice EPSI sequence (TR/TE_eff_ = 900/3.525 ms, field of view = 32 × 32 mm^2^, flip angle of 41°, image size of 12 × 12, in-plane resolution of 2.667 × 2.667 mm^2^, six slices of 3.5-mm thickness covering the whole brain, one average, and scan time of 11 s). The ^13^C hyperpolarized EPSI scan was performed 25 s after the start of the injection time, which was found to be optimal for metabolite production in the mouse brain. To normalize the ^13^C signal of the metabolic substrates and products in the mouse brain, the ^13^C EPSI scan was repeated to image the 6 M ^13^C-labeled urea phantom after optimizing the homogeneity of the magnet field over the phantom volume.

#### In vivo ^13^C CSI

To investigate whether the copolarization of multiple isotope positions, [1-^13^C]pyruvate and [2-^13^C]pyruvate along with [1-^13^C]DHA, could give insights into the compartmentalization of key metabolites in the murine brain, four independent experiments were performed on athymic nude mice (*n* = 4). Following the mouse setup, each mouse underwent the ^13^C hyperpolarized MRI scan 25 s after injecting 250 μl of cohyperpolarized 40 mM [1-^13^C]DHA, 50 mM [1-^13^C]pyruvate, and 88 mM [2-^13^C]pyruvate. The ^13^C hyperpolarized scan was acquired in the coronal orientation using a 2D multivoxel CSI sequence (TR/TE_eff_ = 141.4/5 ms, field of view = 32 × 32 mm^2^, flip angle of 20°, in-plane resolution of 2.667 × 2.667 mm^2^, one slice of 15 mm in thickness covering the whole brain, one average, and scan time of 20.4 s). For each mouse, the ^13^C CSI sequence was repeated to image the ^13^C urea phantom for signal normalization purposes, as previously described.

To investigate brain tumor metabolism, U87 glioma-bearing mice with comparable tumor size (tumor volume ranging from 14 to 20 mm^3^) were injected with 300 μl of cohyperpolarized 40 mM [1-^13^C]DHA and 100 mM[1-^13^C]pyruvate over 10 s. Mice were imaged using the same MRI protocol previously described except for the 5-mm thickness of the CSI, which covered the entire tumor and was centered in it.

### Data analysis

For each set of hyperpolarized ^13^C experiments, data analysis was performed as below.

#### In vivo ^13^C NMR spectroscopic data analysis

First, a Lorentzian apodization of 10 Hz was applied to ^13^C NMR. For each time point, each peak of every spectrum was integrated to generate time varying data, as previously described (*42*). Experimental signal integrals of all metabolites for each hyperpolarized ^13^C experiment (*n* = 3) were quantitatively modeled using a custom Matlab script (Matlab R2021b, The MathWorks). Kinetic rate constants related to the conversion of [1-^13^C]pyruvate to [1-^13^C]lactate (*k*_PL_), [1-^13^C]pyruvate to [1-^13^C]alanine (*k*_PA_), [1-^13^C]pyruvate to [1-^13^C]bicarbonate (*k*_PB_), and [1-^13^C]DHA to [1-^13^C]VitC (*k*_DV_) were calculated using an “inputless” (*42*). The estimated longitudinal relaxation time (*T*_1_) of each metabolite was set to 50 s with a fitting range extended from 10 to 80 s.

#### In vivo ^13^C EPSI data analysis

ROIs were defined on the coronal TSE T_2_-weighted images (Fig. 4A) of a mouse brain. ROIs were drawn symmetrically in the right and left hemispheres of the mouse brain, ensuring their position within the corpus callosum, the largest WM structure (Fig. 4, A to C, red ROIs) in the brain, and the thalamus, which has a GM structure (Fig. 4, A to C; ocean-blue ROIs). For each mouse, T_2_-weighted images and corresponding ROIs were overlaid on the related MRSI data and metabolic maps, which were calculated using the Spectroscopic Imaging, Visualization and Computing (Sivic 0.9) software package (*43*). Before computing the metabolite maps of the metabolites ([1-^13^C]pyruvate and [1-^13^C]DHA) and downstream metabolites ([1-^13^C]lactate and [1-^13^C]VitC), a preprocessing step applied to each hyperpolarized ^13^C spectra per slice [Lorentzian apodization of 5 Hz, first-order and second-order phase, and baseline correction; zero-filling interpolation (ZFP) resulting in an image size of 24 × 24; and in-plane resolution of 1.333 × 1.333 mm^2^] was performed using a custom Matlab script (Matlab R2021b, The MathWorks). For each mouse, an ROI-based analysis was performed on each metabolite map overlaid on the corresponding T_2_-weighted image. The quantified value of each metabolite per brain region was normalized to the quantified value of the 6 M ^13^C-labeled urea phantom, used as a reference.

#### In vivo ^13^C CSI data analysis

ROIs were defined on the coronal TSE T_2_-weighted image (fig. S6) of a mouse brain. ROIs were drawn to ensure their position within the cortical region (CR) of the mouse brain (fig. S6, black ROI) and the thalamus nuclei, which we named DGM (fig. S6, ocean-blue ROIs) to differentiate from the GM of the CR. For each mouse, the T_2_-weighted image and corresponding ROIs were overlaid on the multivoxel CSI data, which were analyzed using the jMRUI (*44*) software package version 7.0 build0.04 (http://jmrui.eu/). A first preprocessing step was applied to each ^13^C spectrum of the hyperpolarized ^13^C MRSI scan (Lorentzian apodization of 3 Hz, first-order and second-order phase, and baseline correction; ZFP resulting in an image size of 36 × 36; and in-plane resolution of 0.889 × 0.889 mm^2^). Quantification of ^13^C NMR spectral peak areas was performed for each spectrum in the CR and DGM brain regions in time domain using the AMARES (Advanced Method for Accurate, Robust, and Efficient Spectral fitting) (*45*) algorithm of jMRUI, which provides the estimated fit for each individual component, the overall spectrum, and the residual line. For each experiment, the mean values of each quantified metabolite per ROI was determined and normalized to the 6 M ^13^C urea phantom. The same analysis was performed for ^13^C MRSI data acquired on a mouse injected only with [1-^13^C]pyruvate and [1-^13^C]DHA to evaluate differences with the metabolite production, especially [5-^13^C]glutamate in the CR and DGM brain regions.

### Statistical analysis

Statistical analysis was performed using GraphPad Prism version 10.0.3 (GraphPad Software, La Jolla, CA, United States). Values are reported as means ± SD for all dataset unless specified otherwise. For slab dynamic, ^13^C MRS experiments (*n* = 3) were used. For the hyperpolarized ^13^C multislice EPSI experiments (*n* = 4), statistically significant differences of the mean metabolite values in the WM and GM brain regions were determined by a two-tailed, paired, Student’s *t* test. The same statistical analysis was applied to the mean metabolite values determined in the CR and DGM brain regions when multivoxel hyperpolarized ^13^C CSI experiments (*n* = 4) were considered. In all statistical analysis, *P* < 0.05 was taken as significant.
