# Supplementary material for: Simultaneous noninvasive quantification of redox and downstream glycolytic fluxes reveals compartmentalized brain metabolism
Source: Sci Adv. 2024 Dec 20;10(51):eadr2058. doi: 10.1126/sciadv.adr2058 (PMC11661454; doi:10.1126/sciadv.adr2058)
Supplement: Supplementary file 1 — Supplementary Text Figs. S1 to S8 Table S1 [file sciadv.adr2058_sm.pdf]

Science Advances
AAAS

Supplementary Materials for

Simultaneous noninvasive quantification of redox and downstream glycolytic fluxes reveals compartmentalized brain metabolism

Saket Patel *et al.*

Corresponding author: Kayvan R. Keshari, rahimikk@mskcc.org

Sci. Adv. **10**, eadr2058 (2024)
DOI: 10.1126/sciadv.adr2058

This PDF file includes:

Supplementary Text
Figs. S1 to S8
Table S1

1. Synthesis of Dehydroascorbic acid

Activated charcoal (7.0 g) was suspended in methanol (250 mL) in a 500 mL Erlenmeyer flask and the charcoal suspension was stirred at room temperature for 1 hour. After an hour, the charcoal suspension was filtered using a Büchner funnel, washed with 30 mL of methanol and dried.

A 5.0 g of [1-^{13}C]ascorbic acid was taken in a 500 mL Erlenmeyer flask and dissolved in 250 mL of methanol followed by the addition of 6.5 g (1.3 x weight of ascorbic acid) of dried activated charcoal powder to the flask. The resulting mixture was then stirred at room temperature overnight (14 hours). After 14 hours, the complete conversion of ascorbic acid to dehydroascorbic acid was confirmed using ^{13}C NMR spectroscopy. After the complete oxidation of ascorbic acid, the reaction mixture was filtered using a Büchner funnel to remove the charcoal and washed with 50 mL of methanol. The resulting filtrate was concentrated on *vacuo* and dried overnight on high vacuum to remove the traces of methanol to afford dehydroascorbic acid (DHA) as a white crystalline semi-solid (4.5 g, 91.09 % yield). The product was characterized using ^{13}C and ^{1}H NMR spectroscopy (600 MHz, Bruker NMR spectrometer) in D_2O and stored at -25 ºC. **^{1}H NMR (600 MHz, D_2O): δ ^{1}H NMR (600 MHz, D_2O) δ** 4.65 (d, *J* = 1.4 Hz, 1H), 4.48 (dd, *J* = 5.4, 2.6 Hz, 1H), 4.17 (dd, *J* = 10.4, 5.4 Hz, 1H), 4.06 (dd, *J* = 10.4, 2.6 Hz, 1H). **^{13}C NMR (151 MHz, D_2O): δ** 173.33, 105.39 (d, *J* = 11.2 Hz), 91.04 (d, *J* = 67.6 Hz), 87.29, 75.94, 72.60 (d, *J* = 1.4 Hz). Melting point of the product was measured to be 29.5±0.6 ℃.

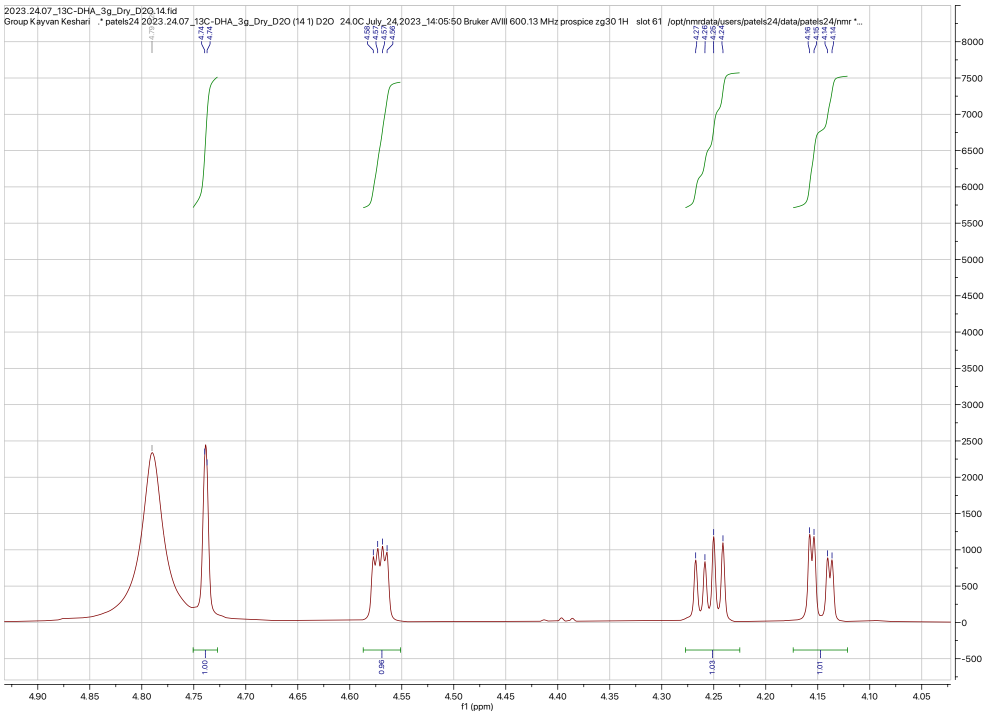

Figure S1: ^{1}H NMR spectrum of [1-^{13}C]DHA in D_2O measured at 600 MHz NMR spectrometer, STP.

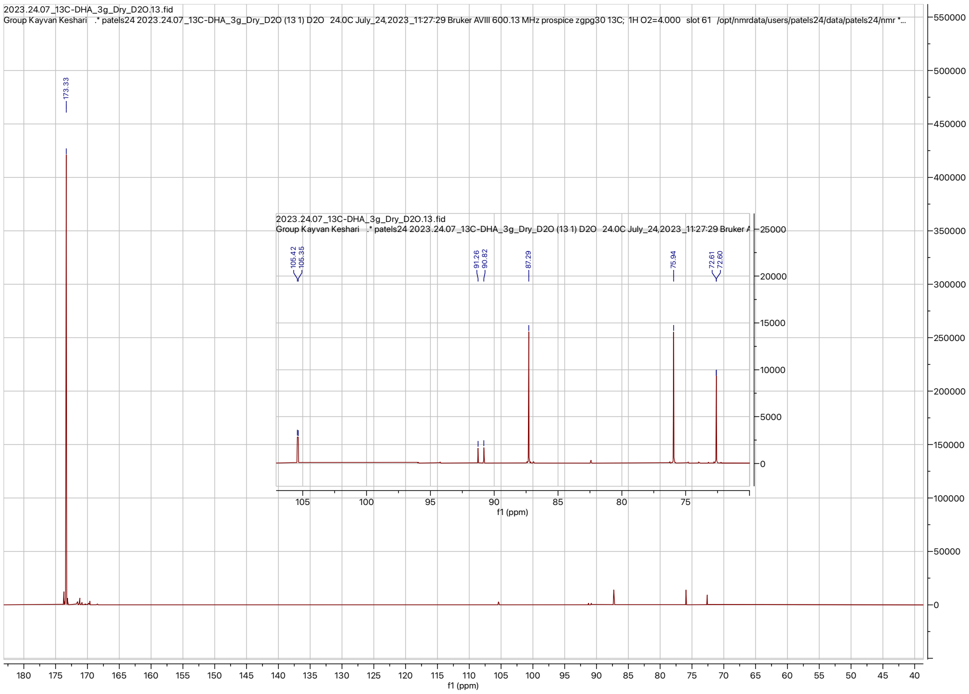

Figure S2: ^{13}C NMR spectrum of [1-^{13}C]DHA in D_2O measured at 600 MHz NMR spectrometer, STP.

2. Stability of DHA

Post demonstrating the DHA monomer as an efficient HP ^{13}C probe for assessing brain redox, the ability of DHA as a glassing solvent for preparing new HP solution with other crucial metabolites to investigate various metabolic pathways (Table S1) was explored. HP probes were dissolved in aqueous DHA or only DHA or in the DHA/PA (40/60; v/v) cocktail to produce the HP formulation consisting of a high substrate concentration and better glassing properties. All the formulations, listed in **Table S1**, created using DHA, aq. DHA or DHA/PA cocktail formed a glass when frozen in liquid N2 which is crucial for getting efficient spectral diffusion and polarization transfer.

Figure S3.

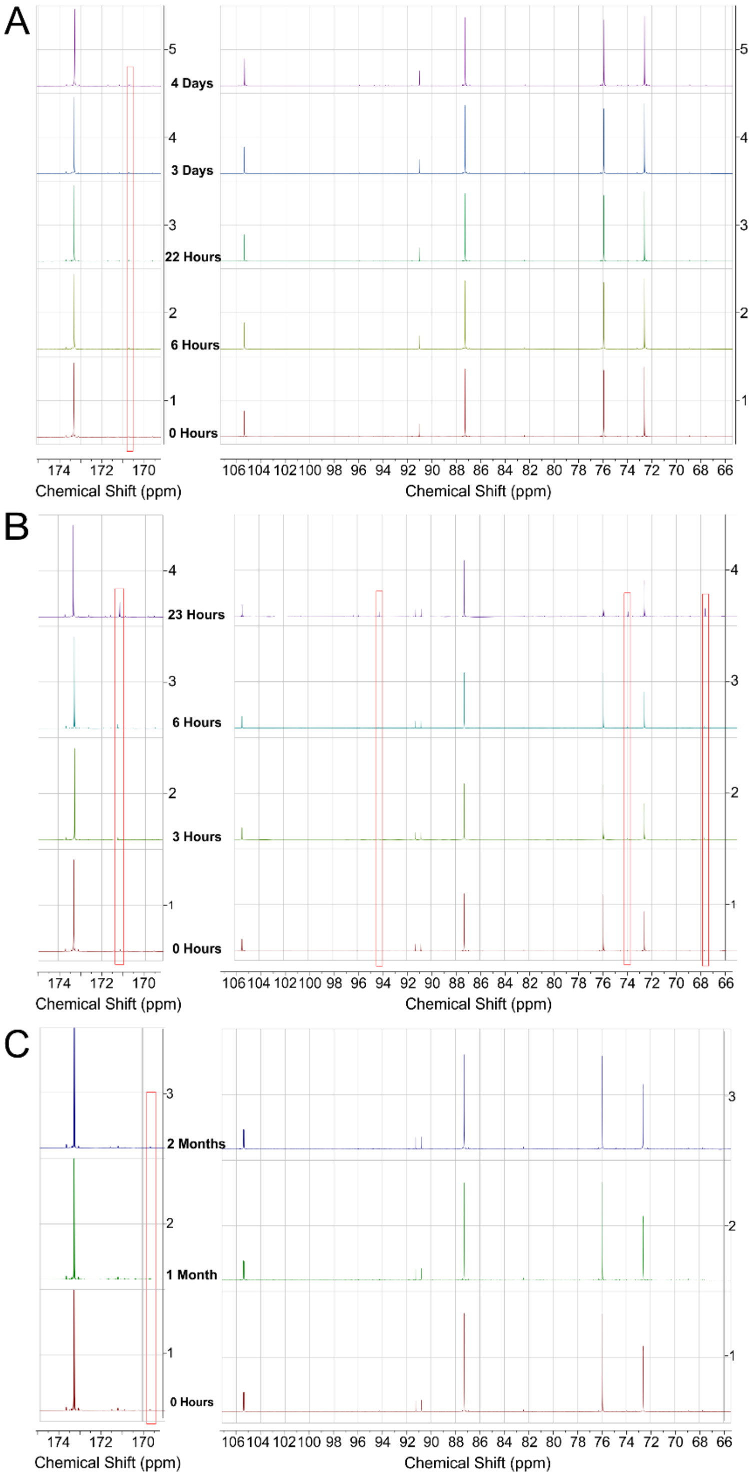

Figure S3. Stability of Dehydroascorbic acid in solution and different temperatures evaluated by ^{13}C NMR spectroscopy. Stability of DHA at (**A**) Solid DHA at room temperature; and (**B**) Aqueous DHA solution at room temperature, and (**C**) DHA solid Frozen at -25 ºC. The formation of impurities via DHA degradation has been highlighted by a rectangular dotted red box.

3. Generality of DHA as a glassing agent and copolarization substrate for HP Studies

To demonstrate the glassing ability of DHA, HP formulation of [2-^{13}C]dihydroxyacetone, [2-^{13}C]glycerate, and [2-^{13}C]fructose in aq. DHA were tested and resulted in significant increase in the ^{13}C polarization as compared to previous work. Formulation of 5.5M [2-^{13}C]Fructose in DHA/H2O provided 2.5x fold increase in ^{13}C polarization compared to previously obtained polarization of 12% for 4M fructose formulation. For [2-^{13}C]dihydroxyacetone, ^{13}C polarization was increased form 16% to ~19.0 % along with the substrate concentration increase of 9.26 M from 8 M. 4M [2-^{13}C]glycerate formulation in DHA/H2O provided the polarization of ~15%. It is evident that DHA as a glassing solvent for preparing HP formulations is very beneficial for not only achieving higher polarization but also eliminating exogeneous glassing solvents. Additionally, DHA can be used both as a glassing solvent as well as a HP ^{13}C substrate providing a possibility of investigating multiple metabolic pathways simultaneously.

Substrate	Metabolic Activity	Reference Formulation	Formulation
2-ketoisocaproic Acid	BCAT activity	KIC/Glycerol (**5 M**)	KIC/DHA (70/30) (**5.907 M**)
2-Ketobutyric Acid	LDH	kBA/(Glycerol/water; 50/50) (**3.4 M**)	kBA/DHA/PA (60/16/24) (**7.08 M**)
3-Hydroxybutyric Acid	Ketone body metabolism	Not Reported	HBA/DHA (70/30) (**7.56 M**)
Choline Chloride	Cellular phospholipid metabolism	ChCl/Water (~**4 M**)	ChCl/DHA/H2O **(4.4 M)**
D-Fructose	Fructolysis	Fructose/water (**4 M**)	Fructose/DHA/H_2O (**5.5 M**)
Glycerate	C2 cycle	Glycerate/ (Glycerol/water; 3/2) (**3 M**)	Glycerate/DHA/H_2O (**4 M**)
Dihydroxy Acetone	Gluconeogenesis	DHAc/Water (**8.0 M**)	DHAc/DHA/H_2O (**9.26 M**)

Table S1: Generality of DHA as a co-solvent and substrate in creating efficient HP formulations for multiple crucial metabolites of interest.

Figure S4 - ^{13}C Polarization Dynamics

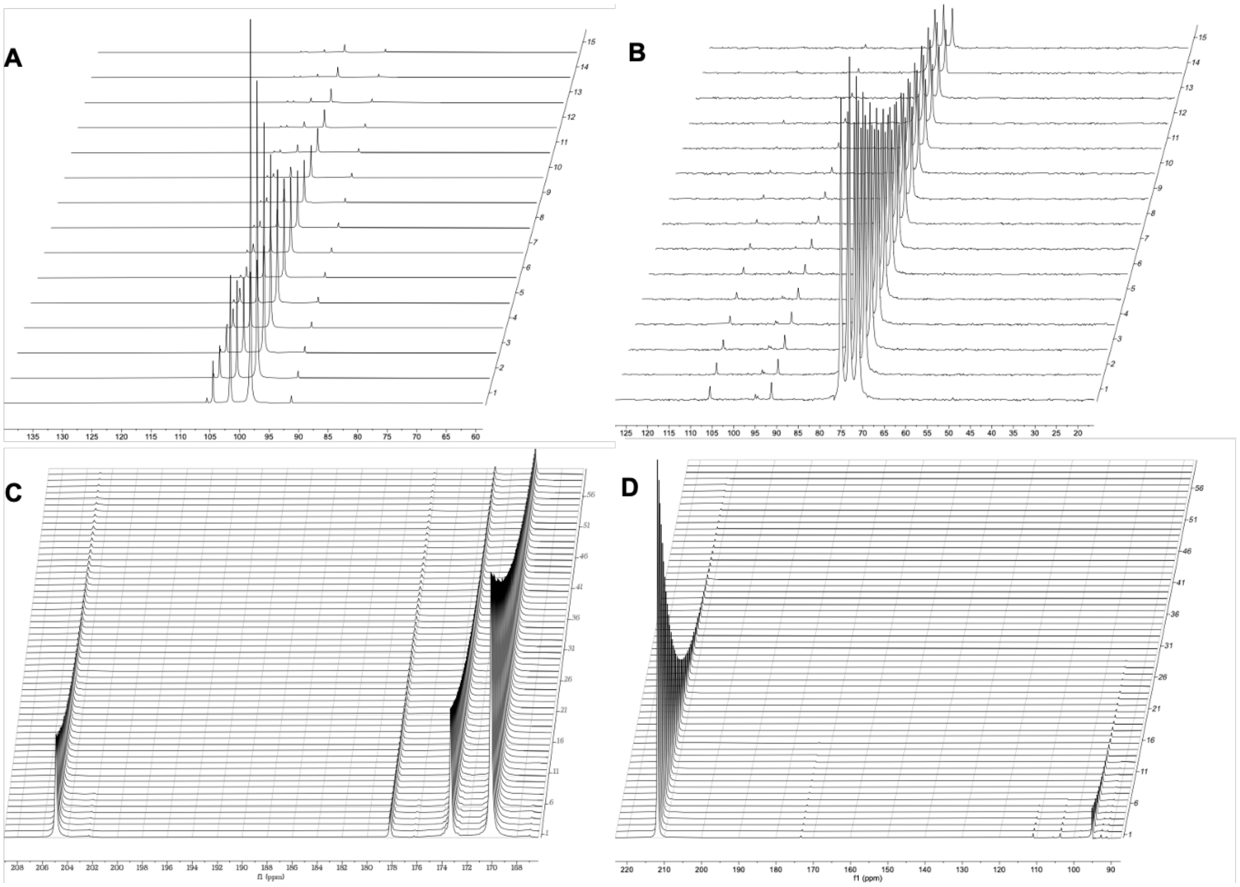

Figure S4. Representative ^{13}C dynamics of HP substrates prepared using DHA as a glassing agent, dissolved in D_2O and dynamic measured at 1T magritek benchtop NMR spectrometer. (**A**) HP [2-^{13}C]Fructose; (**B**) HP [2-^{13}C]glycerate; (**C**) HP [1-^{13}C]pyruvate/[2-^{13}C]pyruvate/[1-^{13}C]DHA; and (**D**) HP [2-^{13}C]DHAc.

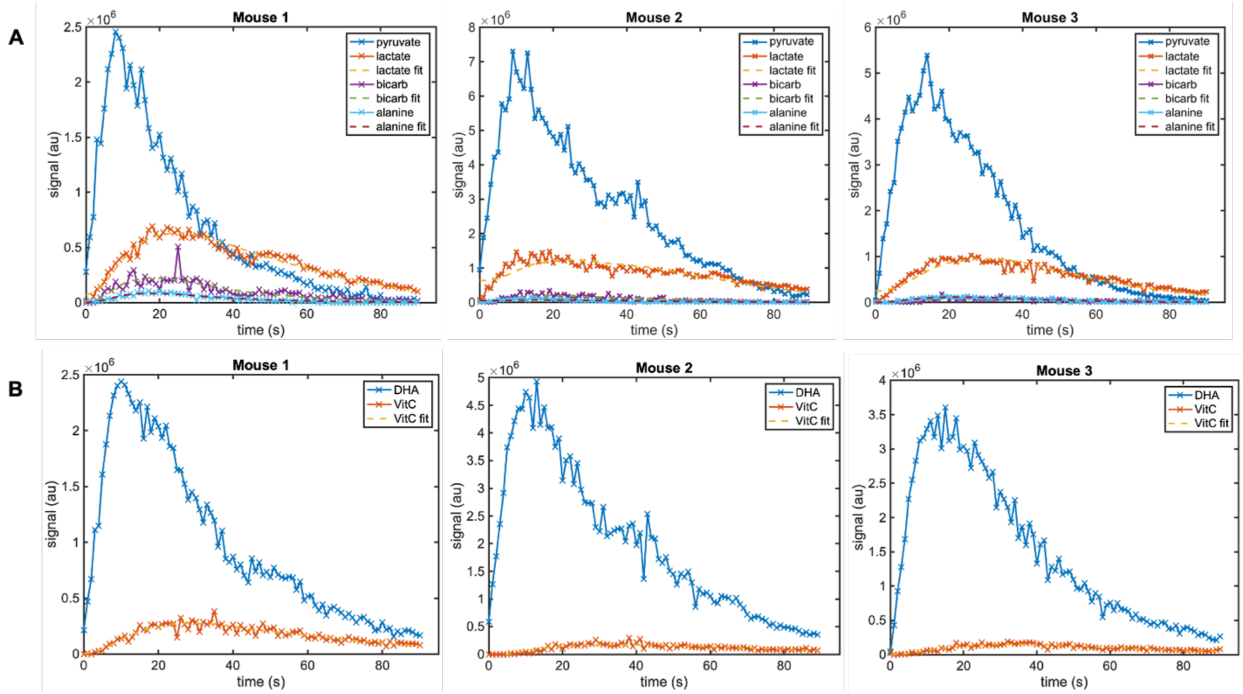

Figure S5. Kinetic fits analyzed from the dynamic HP MRS of HP [1-^{13}C]Pyruvate/[1-^{13}C]DHA in the murine brain. (**A**) Kinetic fits of HP pyruvate conversion to its downstream metabolites- lactate, bicarbonate, and alanine for three replicates. (**B**) Kinetic fits of HP DHA conversion to vitamin C (VitC), n=3 biological replicates.

4. **Co-hyperpolarized [1-^{13}C]pyruvate and [1-^{13}C]dehydroascorbate (DHA) to investigate metabolism in a glioma model**

To simultaneously evaluate glycolytic metabolism and oxidative stress in a disease state using co-polarized **[**1-^{13}C]pyruvate and [1-^{13}C]DHA, orthotopic xenografts of U87 glioblastoma were used. Mice underwent a stereotactic orthotopic implantation of U87 glioblastoma cells in the striatum (caudate putamen). Tumor growth was monitored twice a week through MRI. Once tumors reached at least 3 mm in diameter (approximately a volume of 14.2 mm^3) mice underwent the ^{13}C HP scan. Mice were injected via tail vein over 10s with 300µL of co-hyperpolarized 40 mM [1-^{13}C]DHA and 100 mM [1-^{13}C]pyruvate. Both substrates were co-hyperpolarized for two hours through the SpinLab polarizer (GE, Healthcare) and dissolved in D_2O. MR experiments were performed using a 3T MRI system (Bruker) equipped with a quadrature double-tuned ^{1}H/^{13}C volume coil. The MRI protocol included a T2-weighted sequence for anatomical reference and ^{13}C Chemical Shift Imaging (CSI) sequence (25 x 25 mm^2 field-of-view, 5 mm thickness to cover the entire tumor) run 25s after substrate injection. Spectroscopic data were processed using a custom Matlab script and analyzed in SIVIC software. Normalization for polarization and concentration were performed using the 6M urea phantom. Comparison between metabolite concentrations in tumor lesion and contralateral brain were compared using a two-tailed, paired, Student's *t*-test.

Compared with the contralateral brain, increased mean concentration of both substrates, [1-^{13}C]pyruvate (**Figure 6S, C**) and [1-^{13}C]DHA (**Figure 6S**, **D**), and downstream metabolites, [1-^{13}C]lactate (**Figure 6S, E**) and [1-^{13}C]vitamin C (**Figure 6S F**), was found. A significantly higher lactate production in tumor lesion than healthy contralateral brain shows the sensitivity and feasibility of the method to detect tumor.

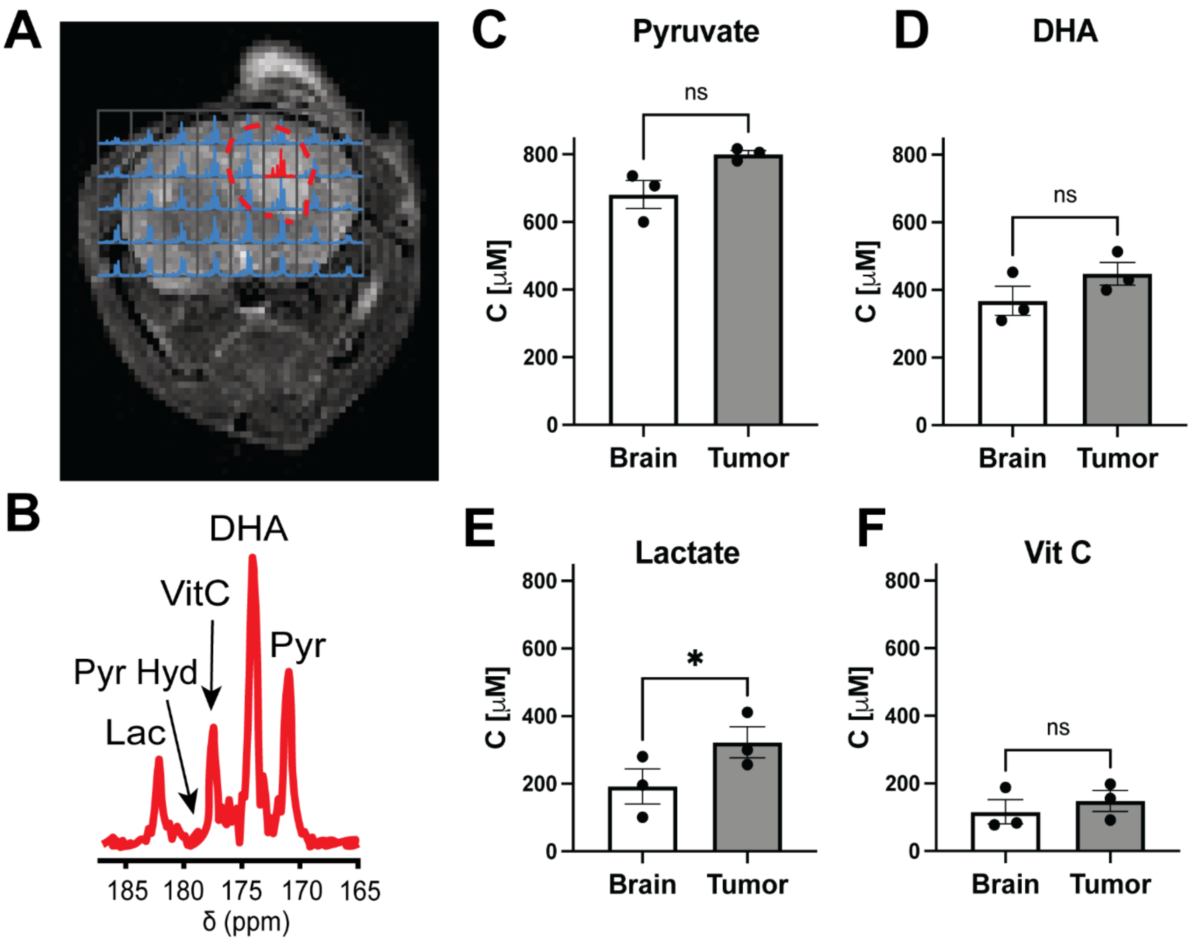

Figure S6. 2D-Multi Voxel Chemical Shift Imaging (CSI) with HP [1-^{13}C]pyruvate and [1-^{13}C]dehydroascorbate (DHA) for the quantification of metabolism in U87 glioma xenograft
(**A**) Overlay of a coronal multi-voxel ^{13}C HP CSI acquisition of a murine brain with U87 xenograft on the corresponding morphological T_2-weighted image. (**B**) Representative ^{13}C HP spectrum (red line) of a voxel in the tumor area showing the main metabolite peaks ([1-^{13}C]pyruvate at 171.1 ppm, [1-^{13}C]DHA at 174 ppm, [1-^{13}C]vitamin C at 178 ppm, [1-^{13}C]pyruvate hydrate at 179 ppm and [1-^{13}C]lactate at 183 ppm). (**C-F**) Quantification of (**C**) [1-^{13}C]pyruvate, (**D**) [1-^{13}C]DHA, (**E**) [1-^{13}C]vitamin C and (**F**) [1-^{13}C]lactate in tumor and contralateral region (Brain).

5. Normalization of ^{13}C-labelled metabolite signal

For each hyperpolarized session, the MRI protocol included the MRSI phantom scan, performed to normalize the ^{13}C signal of each hyperpolarized ^{13}C-labelled metabolite to the [1-^{13}C]urea signal, used as a reference. During animal positioning, a cylindrical phantom (1 cm diameter, 3 cm heigh) containing a 6M [1-^{13}C]urea solution was placed adjacent to the mouse's head (**Figure S7A**) with the main axis parallel to the slice-selection gradient (z-axis of the scanner) in order to cover the full length of the mouse head. Following the ^{13}C hyperpolarized MRI scan on the mouse brain (**Figure SA-C**) and the shim optimization on the phantom, the MRSI scan was repeated to acquire the ^{13}C signal deriving from [1-^{13}C]urea phantom (**Figure S7A,-D-E**). The ^{13}C signal of each hyperpolarized ^{13}C-labelled metabolite was normalized for the integrated peak of the urea

(**Figure S7E**) and corrected for the percentage of the dynamic nuclear polarization determined after dissolution.

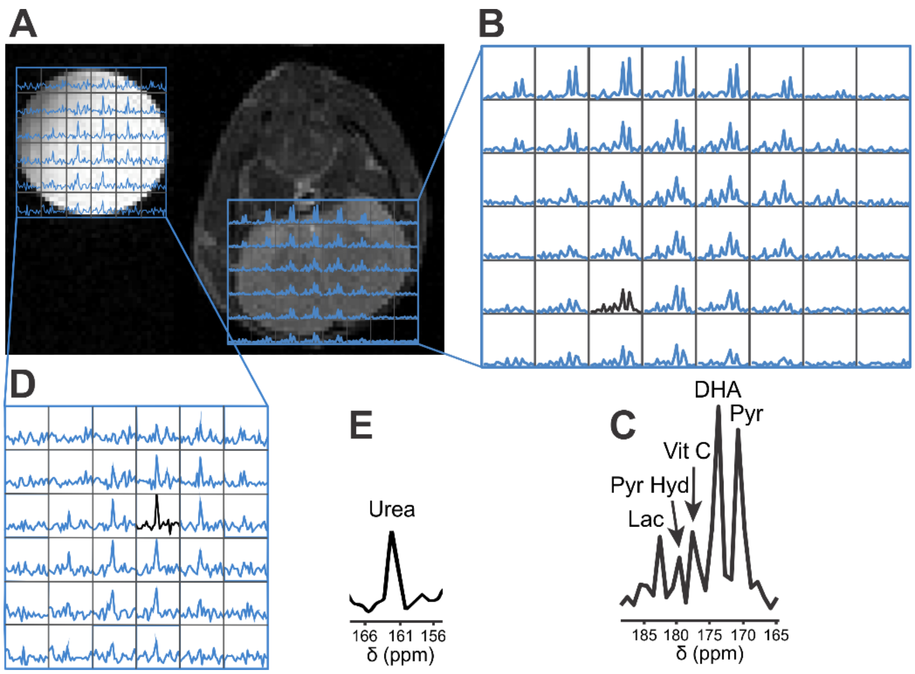

Figure S7. [1-^{13}C]Urea phantom MR scans acquired to normalize the ^{13}C signal of each hyperpolarized metabolite. (**A**) Representative coronal T_2-weighted image of a mouse brain adjacent to the cylindrical phantom containing a 6M [1-^{13}C]urea solution, used as a reference. The overlay of the corresponding ^{13}C MRSI grid from the stack images acquired using the multi-slice echo planar spectroscopic imaging (EPSI) scan is shown for the HP EPSI scan acquired on the mouse brain and the EPSI scan acquired on the phantom during the same MRI session.(**B**) A magnification of the overlayed grid (**A**) on the mouse brain with the ^{13}C EPSI spectra showing the spatial distribution of the substrates and downstream metabolites. (**C**) Representative ^{13}C HP spectrum of the expanded grid (**B**, black line) showing the main metabolite peaks ([1-^{13}C]pyruvate at 171.1 ppm, [1-^{13}C]DHA at 174 ppm, [1-^{13}C]vitamic C at 178 ppm, [1-^{13}C]pyruvate hydrate at 179 ppm and [1-^{13}C]lactate at 183 ppm). (**D**) A magnification of the overlayed grid on the phantom (**A**) showing the spatial distribution of [1-^{13}C]urea signal over the phantom. (**D**) The central [1-^{13}C]urea spectrum of the grid (**-D**, black line) showing the maximum [1-^{13}C]urea signal on the phantom.

4. 6. Matrix-Assisted Laser Desorption Ionization Imaging Mass Spectrometry

Following *in vivo* hyperpolarized MRI, mice were euthanized for MALDI (Matrix-Assisted Laser Desorption Ionization Imaging Mass Spectrometry) imaging mass spectrometry (IMS) to assess the glycolytic metabolism**.** Dissected brains were snap-frozen in 2-Methylbutane (Cat # M0167; Sigma Aldrich, St Louis, MA, USA) place in dry-ice and pre-cooled at -80 °C. Frozen brains were cryo-sectioned (12-m thick) in the sagittal orientation at −18 °C using a Leica CM 1950 Cryostat (Leica Microsystem GmbH, Wetzlar, Germany) and then thaw mounted onto indium tin oxide (ITO, Delta Technologies, Limited – Loveland, CO, USA) coated glass slides and stored at -80°C. Prior matrix deposition with N-(1-naphthyl) ethylenediamine dinitrate (NEDN) in negative ion mode and matrix sprayer HTX TM-Sprayer™ (HTX Technologies LLC, NC, USA), ITO-slides were dried for 30 min under a vacuum chamber. Immediately after, they were loaded on a 7T scimaX-MRMS (Magnetic Resonance Mass Spectrometry) system (Bruker Daltonics Inc, Billerica, USA) equipped with a SmartBeam II laser and a MALDI source. Data were acquired with a spatial

resolution of 80 µm × 80 µm and acquisition time of 2.5 minutes per mm^2. Data visualization and analysis was performed using Scils Lab software (SCiLS GmBH, Bremen, Germany).

5. Multi-voxel Chemical Shift Imaging in vivo with HP [1-^{13}C]DHA and [1-^{13}C]pyruvate

To further confirm our findings on the injection of copolarized HP [1-^{13}C]DHA, [1-^{13}C]pyruvate and [2-^{13}C]pyruvate a 2D multi-voxel Chemical Shift Imaging (CSI) experiment was performed in vivo on an athymic nude mouse by injecting only copolarized HP [1-^{13}C]DHA and [1-^{13}C]pyruvate. The experiment was carried out following the same animal procedures and MRI protocol used for the mice injected with the three probes. Results showing the metabolites peaks detected in the cortical (CR) and deep gray matter (DGM) regions are shown in **Figure 7SB-C**. In both brain regions the [5-^{13}C]glutamate peak at 181.4 ppm is not visible (red arrow). On the contrary, [5-^{13}C]glutamate was detected and quantified in the cortical brain region when mice were injected with the co-polarized three probes (**Figure 5B**).

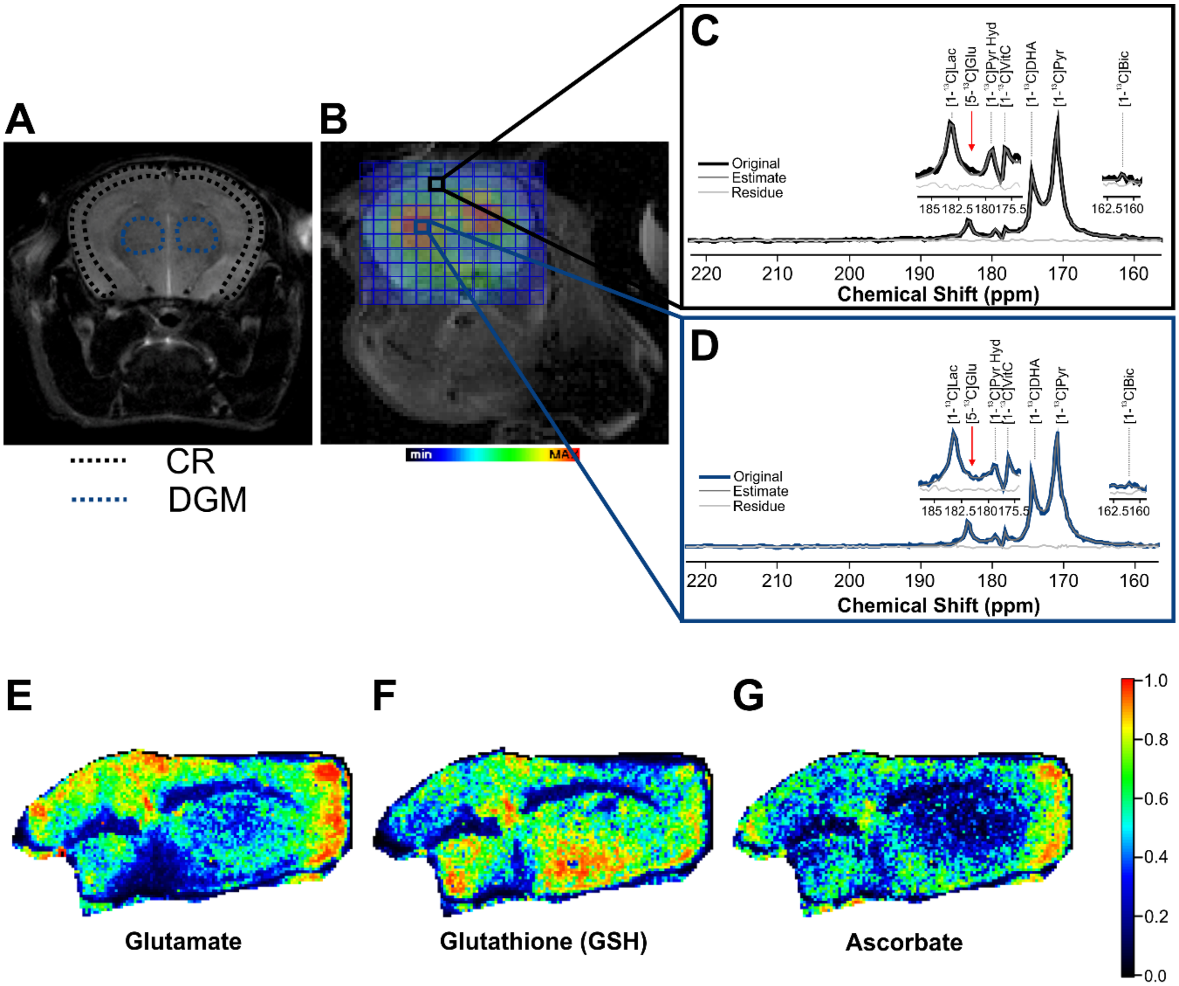

Figure S8 2D multi-voxel Chemical Shift Imaging (CSI) with HP [1-^{13}C]pyruvate and [1-^{13}C] dehydroascorbate (DHA) to infer regional metabolism in a mouse brain.

(**A**) Representative T_2-weighted coronal image of a mouse brain showing the cortical (CR) region-of-interest (ROI) (black dotted line) and the deep grey matter (DGM) ROIs (ocean blue dotted lines)**.** The image was acquired on a mouse brain with in-plane resolution of 102 × 102 μm^2. (**B**) Overlay of a coronal multi-voxel ^{13}C HP MRSI acquisition of a mouse brain in color levels (blue = min, red = max) on the corresponding morphological ^{1}H T_2-weighted image (in grey levels). The color of each voxel of the MRSI grid indicates the total ^{13}C signal in that voxel. (**C**) Representative ^{13}C HP spectrum (black line) of a voxel in the cortical region (CR) of a mouse brain showing the main metabolite peaks ([1-^{13}C]pyruvate at 171.1 ppm, [1-^{13}C]bicarbonate at 161.1 ppm, [1-^{13}C] pyruvate hydrate at 179 ppm, [1-^{13}C]lactate at 183ppm, [1-^{13}C]DHA at 174 ppm and [1-^{13}C]vitamin C at 178 ppm). (**D**) Representative ^{13}C HP spectrum (ocean blue line) of the deep grey matter region (DGM) of a mouse brain showing the main metabolites peaks. The [5-^{13}C]glutamate signal at 181.4 ppm is not visible in both cortical and DGM regions (red arrow; B,C). For both spectra (B,C), a quantitative estimate (dark grey line) obtained using AMARES (Advanced Method for Accurate, Robust, and Efficient Spectral fitting; jMRUI) is shown along with the residual line (light grey line). A magnification of the spectral regions where the metabolites of interest are observed (175.5 – 185 ppm and 160 -162.5 ppm) is shown for each spectrum (C,D). (**E-G**) Heatmaps visualization of glutamate (**E**), glutathione (**F**) and ascorbate (**G**) from a sagittal section of a mouse brain obtained using MALDI-IMS. The heatmaps display the relative levels of each metabolite in the murine brain (red = high level, deep blue = low level).
